# Characterization of ShigETEC, a Novel Live Attenuated Combined Vaccine against Shigellae and ETEC

**DOI:** 10.3390/vaccines8040689

**Published:** 2020-11-16

**Authors:** Shushan Harutyunyan, Irene Neuhauser, Alexandra Mayer, Michael Aichinger, Valéria Szijártó, Gábor Nagy, Eszter Nagy, Petra Girardi, Frank J. Malinoski, Tamás Henics

**Affiliations:** 1 Eveliqure Biotechnologies GmbH, Karl-Farkas-Gasse 22, 1030 Vienna, Austria; Shushan.Harutyunyan@eveliqure.com (S.H.); Irene.Neuhauser@eveliqure.com (I.N.); alexandra.mayer1@gmx.at (A.M.); michael.christoph.aichinger@outlook.com (M.A.); valeria.szijarto@eveliqure.com (V.S.); Gabor.Nagy@eveliqure.com (G.N.); eszter.nagy@eveliqure.com (E.N.); Petra.Girardi@eveliqure.com (P.G.); Frank.Malinoski@eveliqure.com (F.J.M.); 2 CEBINA GmbH, Karl-Farkas-Gasse 22, 1030 Vienna, Austria

**Keywords:** vaccine, *Shigella*, cross-protection, rough, non-invasive, ETEC, heat-stable toxin, heat-labile toxin

## Abstract

**Background**: *Shigella* spp. and enterotoxigenic *Escherichia coli* (ETEC) remain the two leading bacterial causes of diarrheal diseases worldwide. Attempts to develop preventive vaccines against Shigella and ETEC have not yet been successful. The major challenge for a broad Shigella vaccine is the serotype-specific immune response to the otherwise protective LPS O-antigen. ETEC vaccines mainly rely on the heat-labile enterotoxin (LT), while heat-stable toxin (ST) has also been shown to be an important virulence factor. **Methods**: We constructed a combined Shigella and ETEC vaccine (ShigETEC) based on a live attenuated Shigella strain rendered rough and non-invasive with heterologous expression of two ETEC antigens, LTB and a detoxified version of ST (ST_N12S_). This new vaccine strain was characterized and tested for immunogenicity in relevant animal models. **Results**: Immunization with ShigETEC resulted in serotype independent protection in the mouse lung shigellosis model and induced high titer IgG and IgA antibodies against bacterial lysates, and anti-ETEC toxin antibodies with neutralizing capacity. **Conclusions**: ShigETEC is a promising oral vaccine candidate against Shigella and ETEC infections and currently in Phase 1 testing.

## 1. Introduction

Diarrheal diseases represent a significant medical burden worldwide. Shigellae and enterotoxigenic *Escherichia coli* (ETEC) are the two leading bacterial causes of diarrheal manifestations in children under five years of age in endemic regions, travelers to countries with endemic disease, as well as military and civilian personnel deployed to endemic regions [1,2,3,4]. Current reports indicate that the 950 million global diarrheal episodes per year lead to 1.3 million deaths out of which 500,000 affect children under five years of age [5,6]. Shigellae alone contribute with 125 million episodes per year, and the 165,000 cases that occur within children under five years of age result in 55,000 deaths [1,7]. ETEC is responsible for an additional 20,000 fatalities annually [5,6,8,9].

Infections with members of the Shigella genus lead to moderate to severe intestinal syndrome called bacillary dysentery or shigellosis. Natural immunity is serotype specific and protection usually develops over time against the encountered serotype and not against the other approx. 50 serotypes of the four Shigella species. The natural immune response is predominantly directed against the immunodominant O-antigen moiety of the bacterial lipopolysaccharide (LPS). An ideal Shigella vaccine should elicit protection against all prevalent serotypes. Based on the four decades history of Shigella vaccine development, it has been challenging to find a balance between adequate safety and sufficient immunogenicity and therefore protection [7].

ETEC is a mucosal enteric pathogen that has overlapping endemicity with Shigella, and infection occurs via the fecal-oral route, such as in case of shigellosis, in areas with low sanitary infrastructure. This pathogen exerts its pathogenicity via two major endotoxins, the heat-stable (ST) and heat-labile (LT) toxins. ST is a 19 amino acid peptide with high toxicity and poor immunogenicity. LT is a complex macromolecule, consisting of one LTA subunit and five LTB subunits. The LT holotoxin is closely related both structurally and functionally to cholera toxin (CT). ETEC strains may express either LT or ST, but often both. Therefore, a viable vaccine should target both LT and ST. Despite multiple attempts, it has been difficult to generate high titer neutralizing antibodies against ETEC toxins. An oral immunization route would be amenable to deliver sufficiently detoxified, yet ideally immunogenic antigens, a task that has not yet been successfully addressed [2,10]. 

Here we describe the construction and immunological characterization of a live, attenuated, non-invasive Shigella vaccine strain that can elicit serotype-independent protection against Shigellae challenge. The broad protection against heterologous Shigella strains is achieved through removal of the serotype-determining O-antigen component of LPS [11]. The expression of the ETEC proteins LTB and a detoxified ST (generated by a single amino acid mutation) by this vaccine strain aims to address ETEC for a broader coverage of diarrheal pathogens. This vaccine candidate, called ShigETEC is currently in Phase 1 safety and immunogenicity testing.

## 2. Materials and Methods

**Figure 1:**
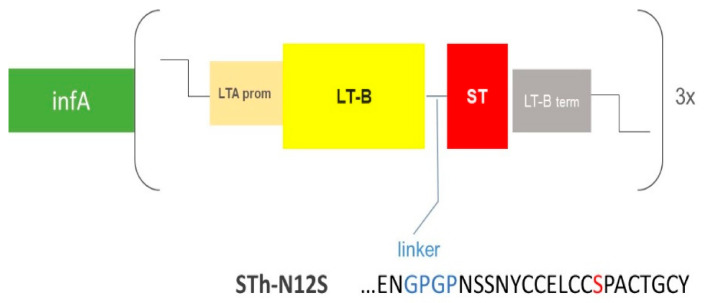
Schematic illustration of the genetic construct carried by the invasion plasmid of ShigETEC. LTB is fused to ST via a GPGP (GlyPro) linker (blue). ST is detoxified by an N12S mutation (red). Expression is driven by the LTA promoter and halted by the LTB termination sequence. The fusion gene is expressed as a 3× tandem repeat. The construct also expresses *infA* as a separate gene with intrinsic promoter and terminator sequences.

Construction of the ShigETEC vaccine strain: The *Shigella flexneri* 2a 2457T was used as parental strain [12]. All genetic manipulations were performed using the λ Red recombinase technique [13,14]. Briefly, mid-log phase bacterial cultures were placed on ice for 5 min and pelleted at 4 °C. Pellets were washed twice with ice-cold ddH_2_O before resuspension in 5% glycerol and transformation with specific constructs targeting the aimed gene locus to be replaced with an antibiotic cassette for subsequent selection. Following selection of correct mutant colonies by PCR, antibiotic cassettes were excised using the helper plasmid pCP20, leaving a scar at the site of the deletions. As scar regions of multiple mutational steps may recombine with each other, integrity of these regions was verified by PCR after each mutagenesis steps. Using this strategy, three loci were deleted: the *rfbF* and *setBA* genes on the chromosome as well as the *ipaBC* cluster located on the large invasion plasmid (IP). Subsequently, a synthetic construct was generated encoding a chimeric LTB-ST_N12S_ (mutant of the natural 19 amino acid peptide where the Asp in position 12 is exchanged to Ser) fusion toxoid product as well as an essential Shigella gene, *infA*. The schematic structure of this construct as well as the sequence of the ST toxoid and the linker region between LTB and the ST_N12S_ toxoid is illustrated in Figure 1. The construct was inserted into an inert site on the IP. Subsequently, the chromosomal *infA* allele was deleted, thereby rendering the IP indispensable for survival. The persistence and integrity of the IP upon repeated in vitro passaging was monitored by PCR, targeting specifically selected regions of the plasmid upon repeated passaging in vitro. 

In vitro cell invasion assay: Invasiveness of the ShigETEC vaccine strain was assessed in vitro using epithelial cells. HeLa cells (ATCC, CCL-2) were grown in Dulbecco’s Modified Eagle Medium (DMEM) with 10% FCS in 24-well plates until confluency. Cells were infected with wild-type *S. flexneri* 2a strain 2457T or the vaccine strain at a multiplicity of infection (MOI) of 80 for 1 h at 37 °C. After incubation, plates were washed twice with phosphate-buffered saline (PBS) and the extracellular bacteria were killed with 50 µg/mL gentamycin (30 min at 37 °C/5% CO_2_). Cells were washed 3 times with PBS before lysis with 1% Triton X-100. Bacteria were quantified by plating onto tryptic soy agar (TSA) plates. Percentages of invasive (intracellular) bacteria was calculated relative to the bacterial numbers in the inoculum determined by plating on TSA.

Serény test (in vivo invasion assay): Female, guinea pigs (4–5 months of age, Charles River) were inoculated in the left eye with ShigETEC and in the right eye with its wild-type parental strain (*S. flexneri* 2457T) at bacterial doses of 10^6^–10^9^ colony forming units (CFU)/eye (suspended in 50 µL of PBS). Two identical independent experiments were performed with 4 animals each (2 animals per dose in total). Animals were monitored for 6 days post-infection and the severity of symptoms (mucopurulent conjunctivitis and keratitis) were scored on a scale of 0–4 with 0 = normal, 1 = exudate, 2 = infiltrated cornea with or without exudate, 3 = blurred cornea with hyperemic conjunctiva, 4 = strongly blurred and deformed cornea with purulent exudate. These studies were performed by Enviroinvest Co, Pécs, Hungary according to the 2010/63/EU and National legislation under approval number BA02/2000-26/20/2019 at the Hungarian authority NÉBIH.

Detection of LTB-ST_N12S_ fusion protein expression: ShigETEC whole cell lysates were prepared from cultures grown to OD_600nm_ 0.5, 2 or overnight (O/N). Expression of the LTB- ST_N12S_ fusion protein was determined by GM1 binding. ELISA plates were coated with 10 µg GM1 (Sigma-Aldrich, Schnelldorf, Germany) per well at 4 °C O/N. Plates were blocked with 2% bovine serum albumin (BSA, Fisher Scientific Austria, GmbH, Vienna, Austria) for 1 h at room temperature (RT). Recombinant LTB (Sigma-Aldrich) was used in serial dilutions as positive control. Lysate of a rough, non-invasive Shigella mutant (Δ*rfbF*Δ*ipaBC*) lacking the LTB-ST_N12S_ construct was used as negative control. Lysates and controls were incubated on plates, and bound LTB was detected with HRP-anti-cholera toxin B subunit (CTB) rabbit polyclonal antibody (Fisher Scientific) and ABTS substrate (Fisher Scientific).

Animal efficacy studies: To measure the efficacy of the ShigETEC vaccine, groups of five 6-week-old, female BALB/c mice (Charles River) were vaccinated 3x bi-weekly with ShigETEC intranasally (i.n.) (10^8^ CFU bacterial suspension in 50 µL PBS/animal). Four weeks after the last vaccination, mice were challenged with minimal lethal doses of either *Shigella sonnei* strain 598 (9 × 10^6^ CFU) or *Shigella flexneri* 6 strain 542 (1.2 × 10^7^ CFU) [11]. Minimal lethal doses used were determined in pilot experiments. Survival was monitored for 14 days post challenge. All animal studies were conducted by Fidelta Ltd. (Zagreb, Croatia) in accordance with the 2010/63/EU and National legislation under institutional ethics committee approval number CAREZG_07-10-91_011. Approval number from Ministry of Agriculture, Republic of Croatia is KLASA: UP/I-322-01/15-01/101, URBROJ: 525-1070255-18-4.

Determination of systemic and mucosal antibody levels: To monitor ShigETEC-induced systemic IgG and mucosal IgA responses, mice were vaccinated 3 times i.n. with 10^8^ CFU of the vaccine strain, and serum was taken two weeks after the first and second vaccination and four weeks after the last immunization. Bronchoalveolar lavages (BAL) were performed terminally 14 days after challenge in survivals. Specific IgG and IgA antibody levels were measured from sera or BAL, respectively, against ShigETEC lysate (prepared from overnight cultures of ShigETEC with 1 × 10^7^ CFU/well) or recombinant LTB (Sigma-Aldrich, 100 ng/well) coated on ELISA plates. Biotinylated ST (synthesized by PepScan) was coated at 100 ng/well on streptavidin pre-coated plates (Fisher Scientific). IgG was detected with peroxidase AffiniPure F(ab’)_2_; Fragment Goat Anti-Mouse IgG (H+L) (Jackson ImmunoResearch, Ely, Cambridgeshire, UK), and IgA was detected with peroxidase conjugate goat anti-mouse IgA (Sigma-Aldrich) and ABTS substrate (Fisher Scientific).

ELISA-based LT neutralization assay: Serum from mice vaccinated three times with ShigETEC i.n. was incubated at 2-, 10- or 50-fold dilution with 10, 25, 50 or 100 ng of LT. The amount of LT that remained free from antibody binding was measured with GM1-coated ELISA plates as described above using anti-cholera toxin beta antibody for detection.

Cell-based LT and ST neutralization assay: T84 human colon epithelial cells (ATCC) were seeded in 24-well plates in 1 mL DMEM/F12 (5% FCS, P/S) and grown until confluency. Medium was changed one day before the experiment. Cells were washed 3 times with medium (DMEM/F12 without FCS, P/S) and pre-incubated with medium containing 1 mM 3-3-isobutyl-1-methylxanthine (IBMX, Sigma-Aldrich). 5 ng recombinant LT or synthetic ST (PepScan) were pre-incubated with serum from mock or ShigETEC vaccinated mice (pools of individual serum samples with high LTB titers after 3x i.n. immunization, or with high ST titers after 3x i.p. vaccination with 200 µg recombinant LTB-ST_N12S_ protein). After pre-incubation of toxin and serum, the mix was transferred to T84 cells and incubated at 37 °C (5% CO_2_) for 3 h. Supernatants were removed, and cells lysed with 0.1 M HCl/1% Triton X-100 at RT. Cell lysates were centrifuged and supernatants were assessed for LT-induced cAMP or ST-induced cGMP by using direct cAMP or cGMP ELISA kits (Enzo Life Sciences, Lausen, Switzerland), respectively, according to manufacturer’s instructions.

Expression of ST mutants in *Escherichia coli*: Generation of constructs: pET24a(+)-pre-pro-ST construct was generated by amplifying the pre-pro-ST gene from ETEC H10407 strain using NdeI-ST-prev (5′-CCCCGATATACATATGAAAAAATC-3′) and ST-BamHI-pfw (5′-TCGCGGATCCTTAATAGCACCCGGTAC-3′) primers and inserting into pET24a(+) expression vector in between BamHI and NdeI restriction sites. pET24a(+)-pre-pro-ST_N12S_ was generated by site directed mutagenesis of the pET24a(+)-ST vector, using ST-Mut-P-fw (5′-AAGCAGGagaACAACACAATTCAC-3′) and ST-Mut-P-rev (5′-GTACCGGGTGCTATTAAGGATC-3′) primers. ST sequences in the vectors were confirmed using Sanger Sequencing. Both vectors, as well as pET24a(+) empty vector were transformed into DE3-Tuner expression cells. ST and ST_N12S_ peptide expression: Overnight cultures of DE3-Tuner cells with pET24a(+), pET24a(+)-pre-pro-ST or pET24a(+)-pre-pro-ST_N12S_ vectors were diluted and grown to OD_600nm_ 1 at 37 °C at 200 rpm in RPMI containing 1% Casamino Acids and 25 µg/mL Kanamycin. Thereafter, expression of peptides was induced using 1 mM IPTG at 37 °C for 4 h at 250 rpm. Cells were centrifuged at 5000 rpm for 10 min and the culture supernatant was collected and filtrated using 0.2 μm cellulose-acetate sterile filter. 

Verification of ST peptide expression and detoxification of the ST_N12S_ peptide: T84 human colon epithelial cells (ATCC) were seeded in 24-well plates in 1 mL DMEM/F12 (5% FCS, P/S) and grown until confluency. Medium was changed one day before the experiment. Cells were washed 3 times with medium (DMEM/F12 without FCS, P/S) and pre-incubated with medium containing 1 mM IBMX. Synthetic ST (PepScan) dilutions were prepared at 100 ng, 25 ng, and 5 ng in DMEM/F12 and incubated on T84 cells at 37 °C/5% CO_2_ for 1 h. Additionally, filtered culture supernatants after ST or ST_N12S_ peptide expression from *E. coli* cells were tested at 200 µL/well. After incubation, supernatants were removed, and cells lysed in 0.1 M HCl/1% Triton X-100. Cell lysates were centrifuged, and supernatants assessed for cGMP induction by direct cGMP ELISA (Enzo Life Sciences) according to manufacturer’s instructions.

## 3. Results

### 3.1. Rational Design and Generation of the ShigETEC Vaccine

The fully sequenced *Shigella flexneri* strain 2457T (Genbank accession#: ADUV00000000.1) harboring the ~200 Kbp (140 MDa) large invasion plasmid was used as the parental strain for the generation of the vaccine strain [12]. To remove the serotype-determining and dominant antigen, the wild-type parental strain was rendered rough—that is lacking expression of the LPS O-antigen—by deletion of the *rfbF* gene from the chromosome. With the aim to generate a non-invasive vaccine strain for oral use, two genes involved in the function of the type III secretion system, *ipaB* and *ipaC*, were deleted from the invasion plasmid (IP). Moreover, to reduce the risk of reactogenicity of the vaccine, the *S. flexneri* specific putative enterotoxin ShET-1, an AB_5_ toxin similar to cholera toxin and LT [15], was also removed by deletion of the genes *setBA* from the chromosome, a step that also eliminated the virulence factor Pic that is encoded at the same locus on the complimentary DNA strand. 

To provide additionally ETEC coverage for the vaccine, a synthetic fusion gene was constructed, which encodes a chimeric LTB-ST_N12S_ fusion toxoid. The LTB subunit is not toxic in the absence of the LTA subunit of the LT holotoxin, while the ST peptide is inherently toxic. Therefore, the N12S mutation was introduced into the ST gene. This mutation had been shown to eliminate toxicity while retaining antigenicity of ST [16]. To ensure higher expression levels, a triple tandem of the LTB-ST_N12S_ fusion protein gene was inserted into the IP. The insert was supplemented with the *infA* gene, a single copy essential gene [17], which was subsequently removed from the chromosome to achieve invasion plasmid stabilization [18] (schematic illustration in Figure 1). 

The resulting strain is designated *Shigella flexneri* 2457TΔ*rfbF*Δ*ipaBC*Δ*infA*Δ*setBA*::*infA*-3x[LTB-ST_N12S_], or ShigETEC. To demonstrate that all intended genetic manipulations (summarized in Table 1) were successfully accomplished in the ShigETEC strain, all expected phenotypic changes were verified by PCR and sequencing.

### 3.2. Phenotypic Characterization of ShigETEC

#### 3.2.1. ShigETEC Expresses Rough Lipopolysaccharide

**Figure 2:**
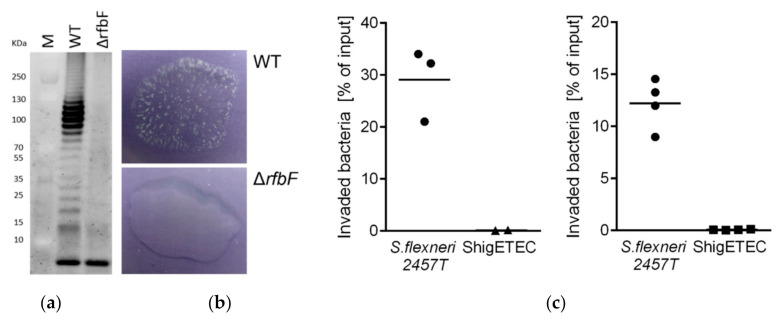
(**a**) SDS-PAGE gel image of separated LPS from Shigella flexneri 2457T wild-type (WT) and *Shigella flexneri* 2457TΔ*rfbF* mutant following staining with Pro-Q^®^ Emerald 300 Lipopolysaccharide Gel Stain Kit. The lowest band represents the lipid A-core molecules, while the upper ladder-like pattern is the LPS molecule with various number of O-antigen repeating units. (**b**) Agglutination assay with *Shigella flexneri* 2457T wild-type (WT, top panel) and its isogenic Δ*rfbF* mutant (bottom panel) using rabbit anti-*Shigella flexneri* 1–6 serum. (**c**) HeLa cells were infected with the wild-type parental *Shigella flexneri* 2a 2457T or ShigETEC at a MOI of 80. Percentage of intracellular (invaded) bacteria relative to the inoculum was determined by CFU calculations after plating. Data are shown from two independent experiments.

As a result of *rfbF* deletion, complete loss of O-antigen was demonstrated by the lack of O-antigen ladder on a silver-stained gel characteristic of LPS preparations from wild-type Shigella strains (Figure 2a). The lack of agglutination of the vaccine strains with an anti-*Shigella flexneri* typing serum confirmed the lack of O-antigen expression (Figure 2b).

#### 3.2.2. ShigETEC Is Non-Invasive and Avirulent

Invasion of human epithelial cells is an inherent characteristic of Shigella and requires the function of the Type III secretion apparatus. Deletion of the *ipaB* and *ipaC* is expected to result in loss of invasive capacity of ShigETEC. This was confirmed in an in vitro invasion assay using HeLa (human epithelial) cells. While the parental wild-type strain was able to invade the cells, ShigETEC completely lost its invasive capacity (Figure 2c).

The Serény test is the gold standard in vivo model to assess the virulence of Shigella strains and is widely used to confirm attenuation before clinical testing in human volunteers [19]. It is performed by inoculation of bacterial suspension in the eye of guinea pigs with virulent Shigella strains causing severe keratoconjunctivitis. To test the virulence of ShigETEC in this model we performed ocular inoculation of guinea pigs with ShigETEC or its wild-type parent strain (*S. flexneri* 2a 2457T). We found that the parental wild-type strain induced keratoconjunctivitis with severity scores proportional to the inoculum size, while the vaccine strain was completely avirulent even at the highest bacterial inoculum (Table 2).

In summary, in vitro and in vivo assays have proven the non-invasiveness and avirulence of ShigETEC. 

#### 3.2.3. ShigETEC Expresses Detoxified ETEC Toxin Antigens

**Figure 3:**
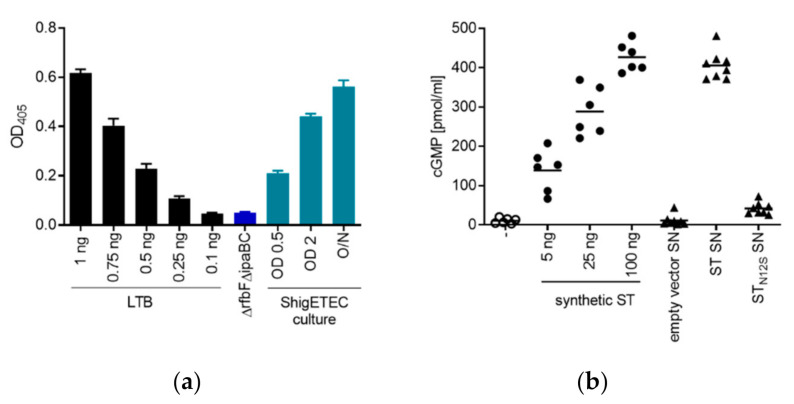
Expression of detoxified ETEC antigens by ShigETEC. (**a**) ShigETEC whole cell lysates were tested for the expression of LTB-ST_N12S_ by binding to the LTB receptor, GM1 in ELISA. Bound LTB was detected by anti-CTB antibody. Expression level of LTB-ST_N12S_ was compared to serially diluted LTB (black bars). A rough, non-invasive Shigella mutant (Δ*rfbF*Δ*ipaBC*) lacking the LTB-ST_N12S_ fusion construct was used as negative control (blue bar). (**b**) Wild-type ST and its N12S mutant were generated recombinantly in *E. coli*. Supernatants (SN) of the cultures were used to stimulate T84 human epithelial cells and ST-induced cGMP production was measured by ELISA. Indicated amounts of synthetic ST were used as positive control. SN from bacteria carrying empty vector served as negative control. Triplicate measurements from two independent experiments were combined.

Expression of the fusion protein of LTB–ST_N12S_ was demonstrated and quantified by ELISA (based on GM1 binding) using lysates of ShigETEC cultures collected in different growth phases. The fusion toxoid was expressed at all growth phases with higher expression upon increasing bacterial concentration (Figure 3a). Specificity of the detection was shown using lysates from a Δ*rfbF*Δ*ipaBC* mutant that did not carry the chimeric toxin antigen (Figure 3a). 

Complete detoxification of the ST_N12S_ mutant was demonstrated by the lack of cGMP induction in T84 human epithelial cells after exposure to supernatants of *E. coli* cultures expressing recombinant wild-type ST or ST_N12S_ (Figure 3b).

### 3.3. ShigETEC Vaccination Provides Serotype-Independent Protection against Shigella Challenge

**Figure 4:**
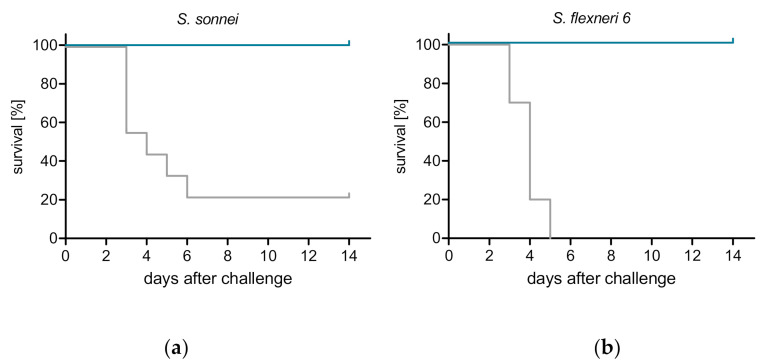
ShigETEC induces protection against lethal challenge with heterologous Shigella strains. Mice were vaccinated 3 times i.n. with 10^8^ CFU ShigETEC (blue line) or buffer (grey line). Four weeks after the last vaccination, mice were challenged i.n. with lethal doses of (**a**) *Shigella sonnei* (9 × 10^6^ CFU) or (**b**) *Shigella flexneri* 6 (1.2 × 10^7^ CFU). Survival was monitored for 14 days. Data from two independent experiments with a total of 10 mice per group are shown.

Due to the lack of appropriate diarrheal models in small laboratory animals, Shigella vaccines are typically evaluated for efficacy in the mouse lung model of shigellosis [20]. The vaccine potential of ShigETEC was tested in this model upon intranasal immunization of mice three times with two-week intervals. Four weeks after the last immunization animals were infected intranasally with lethal doses of wild-type Shigella strains: *S. flexneri* 6 or *S. sonnei* (minimal lethal doses were determined in pilot studies, data not shown). Vaccination with ShigETEC resulted in 100% protection against both of these heterologous serotype strains (Figure 4).

### 3.4. ShigETEC Vaccination Induces Systemic and Mucosal Antibody Responses against Shigellae and ETEC Toxins

**Figure 5:**
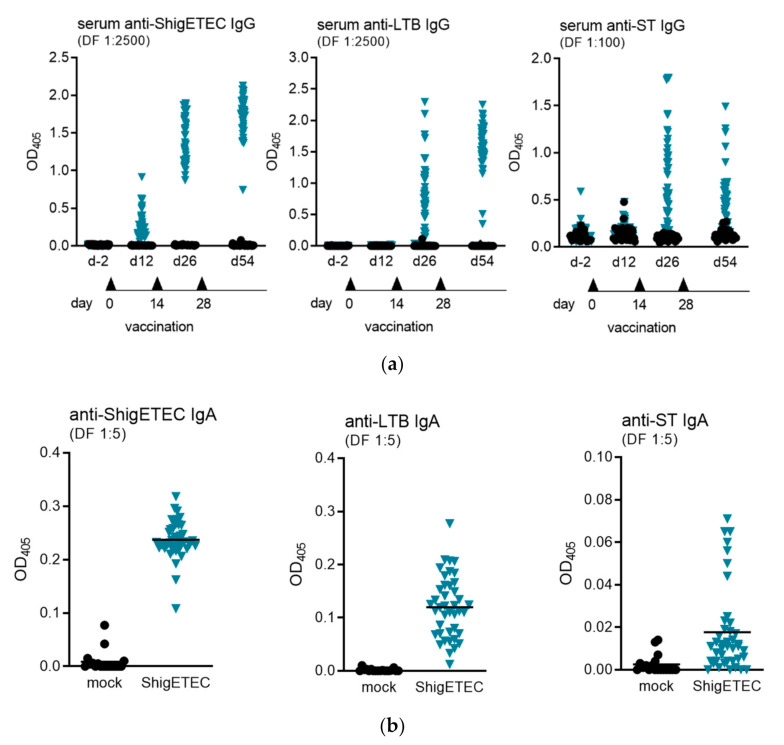
Detection of serum IgG and mucosal IgA antibodies induced upon ShigETEC vaccination. (**a**) Mice were vaccinated 3 times i.n. with 10^8^ CFU ShigETEC. Specific IgG antibody levels were evaluated against indicated antigens in serum obtained 4 weeks after the last vaccination using the indicated serum dilutions in ELISA. Symbols represent averages of duplicate measurements of sera from individual mice (43 mice per group) from three independent vaccination experiments. (**b**) Mice were vaccinated 3 times i.n. with 10^8^ CFU ShigETEC and challenged with lethal doses of either *S. sonnei* or *S. flexneri* 6 four weeks after the last vaccination. Bronchoalveolar lavages (BAL) were taken two weeks after the challenge. Specific IgA antibody levels were evaluated against the indicated antigens using the indicated serum dilutions in ELISA. Symbols represent averages of duplicate measurement of BAL samples from individual mice from three independent vaccination experiments with 18 and 41 mice per group for mock and ShigETEC, respectively.

The antibody response to ShigETEC vaccination was evaluated in mice after 1-, 2- or 3-time intranasal immunization(s). To evaluate systemic antibody responses against Shigella and the ETEC antigens LTB and ST, we measured serum IgG antibodies by ELISA. IgG levels against ShigETEC lysate were detected already after the first vaccination, which increased with the number of vaccinations (Figure 5a, left panel). Antibodies against LTB and ST were detectable only after two vaccinations and with higher variations between individual animals (Figure 5a, middle and right panel). Anti-ST IgG antibody levels were expectedly low, however were clearly induced after three immunizations in 44% of animals with levels 4-fold and in 83% of animals with levels 2-fold over individual pre-immunization levels. 

Bronchoalveolar lavages (BAL) were collected from animals after three times intranasal immunization and subsequent challenge with heterologous Shigella strains two weeks after the challenge. Mucosal IgA antibody responses were measured against ShigETEC lysate, LTB and STAs not only anti-ShigETEC, but also anti-LTB and anti-ST IgA antibodies were detectable in BAL, we concluded that a specific adaptive IgA response was induced by the vaccination. (Figure 5b). In accordance with the history that ST is a poor antigen, induction of anti-ST IgA was low and could only be detected in 17% of animals. 

### 3.5. ShigETEC Vaccination Induces Neutralizing Anti-ETEC Toxin Antibodies

**Figure 6:**
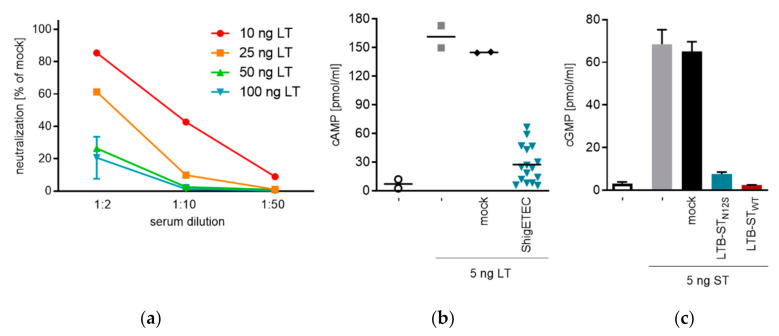
Toxin neutralizing capacity of mouse sera induced by ShigETEC vaccination. (**a**) Mice were vaccinated 3 times i.n. with 10^8^ CFU ShigETEC. Serum at indicated dilutions was incubated with indicated amounts of LT, and LT-binding to GM1 coated plates was measured. Bound LT was detected with a polyclonal anti-cholera toxin antibody. (**b**) Sera from individual mice (3 times i.n. vaccinated with 10^8^ CFU ShigETEC (blue symbols) or buffer (mock, black symbols) were pre-incubated with the 5 ng LT. LT induced cAMP release was measured in T84 human colon epithelial cells. (**c**) 5 ng synthetic ST was pre-incubated with pooled serum from mice vaccinated i.p. with LTB-ST_N12S_ (blue bar) or LTB-ST_WT_ (red bar) protein or vehicle (mock). ST-induced cGMP release was measured in T84 cells.

In order to provide protection against ETEC, a vaccine should elicit anti-LT and anti-ST antibodies with toxin neutralizing capacities. After three times intranasal immunization with ShigETEC we observed induction of anti-LTB and anti-ST IgG in serum. The ability of the vaccine-induced antibodies to block binding of LT to its receptor GM1 was tested in a cell-free ELISA-based assay. We detected serum- and LT-concentration dependent inhibition (Figure 6a). Serum antibodies were also able to inhibit the LT-induced cAMP release from T84 human colon epithelial cells (Figure 6b). Since antibody levels against ST were detectable but low in serum after intranasal vaccination with ShigETEC (Figure 5), no ST-neutralization could be observed with such serum. To prove that the LTB-ST_N12S_ fusion protein as expressed by ShigETEC (Figure 3a) could potentially raise anti-ST neutralizing antibodies, we performed parenteral vaccination with recombinant LTB-ST_N12S_ or LTB-ST_WT_ fusion proteins, which induced high anti-ST IgG levels in serum. These serum antibodies could completely neutralize ST-induced cGMP release in T84 human colon epithelial cells (Figure 6c). Importantly, antibodies raised against LTB-ST_N12S_ were capable of neutralizing wild-type ST in the same manner as antibodies raised against LTB-ST_WT_ confirming that antibodies raised against the mutant ST are functional and can neutralize wild-type ETEC ST.

These data confirm that the LTB-ST_N12S_ as expressed from the invasion plasmid of ShigETEC can induce an antibody response capable of efficiently neutralizing both LT and ST of ETEC.

## 4. Discussion

The ShigETEC vaccine approach to preventing Shigellae and ETEC infections challenges two long-lasting dogmas in vaccine development against Shigella. First, that immunity to Shigella can only be provided by the serotype-determining and dominant LPS O-antigen as the major protective antigen against Shigella [21]. Second, that invasiveness of the Shigella vaccine strains is considered as a prior requirement to develop efficacious oral vaccines [7,22]. However, it has been previously shown that rough Shigella vaccine strains are able to induce serotype-independent protection against lethal Shigella challenge irrespective of the presence of the invasion plasmid that is necessary for invasiveness [11]. Moreover, the only effective Shigella vaccine used in field studies, VADIZEN (developed in Romania in the 1960’s and 70’s), was based on a Shigella *flexneri* 2a strain, T32-Istrati, which was non-invasive [23,24]. Later research identified the genetic basis of this non-invasiveness as the result of a ~80 Kbp deletion in its invasion plasmid that affected virG, virB, icsB, the complete mxi gene cluster as well as the invasion plasmid antigens (Ipa) B, C, D and A [25]. It is likely, that it was the non-invasive phenotype, that allowed the VADIZEN vaccine to be used at high doses, up to 2 × 10^11^ CFU, without reactogenicity. VADIZEN was reported to induce protection after 5 immunizations with 86% efficacy against acute dysentery in children [23,24]. Interestingly, while retaining the LPS O-antigen of the parent strain, the VADIZEN vaccine demonstrated protection against heterologous Shigellae strains, most likely due to the high doses given in 3-day interval. This repeated exposure might have induced antibodies against minor antigens conserved among Shigella serotypes and species [11]. 

Based on these previous studies, we engineered ShigETEC to be non-invasive to epithelial cells and devoid of LPS O-antigen. Here, we provide evidence that ShigETEC is able to induce serotype- and species-independent high level of protection against Shigella challenge in the mouse lung shigellosis model, widely used to assess efficacy of Shigella vaccines [20]. 

The removal of the immunodominant LPS O-antigen allows the exposure of minor antigens conserved among Shigella serotypes and species which skews the immune response toward a general cross-protective Shigella immunity. To induce a broad immune response against minor antigens it is essential for ShigETEC to keep as many potential antigens as possible. Since a number of these antigens are expressed on the IP, it is desirable to retain an intact invasion plasmid except for the attenuating mutations we intentionally introduced to reduce virulence. However, it is known that the invasion plasmid of Shigella has the tendency to be lost completely or partially during in vitro culture [18]. This spontaneous partial plasmid loss or rearrangement is mediated by Shigella’s own recombination system and was demonstrated to be induced by the expression of the virulence genes at 37 °C [26]. It has been hypothesized that this instability of the IP is advantageous for Shigella for the survival outside the host, by allowing conservation of energy that would otherwise be directed towards virulence plasmid maintenance [27]. To ensure retention of the invasion plasmid of ShigETEC and all of its antigens, some of these shown to be protective (e.g., IpaD, IpaB [28,29] the modified invasion plasmid has been stabilized by transposing the essential gene *infA* from the chromosome to the invasion plasmid. This prevents the potential loss of both the retained invasion plasmid antigens and the LTB-ST_N12S_ heterologous ETEC antigen, which was introduced to the plasmid to broaden the protective range of the vaccine to include ETEC. Importantly, this stabilization also ensures that no revertants expressing the wild-type plasmid and thus becoming virulent can emerge in ShigETEC vaccinated individuals.

ETEC pathogenesis is driven mainly by the two diarrhoeagenic toxins: LT (heat labile) and ST (heat stable), and it is the notion in the field that antibodies neutralizing these toxins can prevent the diarrheal symptoms [30]. While both toxins are relatively well-conserved across isolates, and LT is shown to be protective, at least one quarter of ETEC isolates do not express LT, and the most severe forms of ETEC infection are associated with ST-expressing strains [31,32,33]. The challenge of using ST as a vaccine antigen is that it is a small peptide of 19 aa and consequently poorly immunogenic. Moreover, there are limited options to introduce detoxifying mutations without losing the native conformation that is necessary for the induction of neutralizing antibodies. Based on thorough analyses published by Taxt and colleagues [16,34], we decided to use the N12S mutant of ST, which was shown to be non-toxic and still able to induce functional, i.e., toxin-neutralizing antibodies. Importantly, this mutant is not associated with the generation of antibodies cross-reactive with the human uroguanylin [34]. It has been shown that fusing LTB to ST reduces ST toxicity [35,36] while, at the same time, it is expected to increase its immunogenicity. LTB, which is not toxic by itself, possesses potent mucosal adjuvant property [32]. ShigETEC expresses the LTB-ST_N12S_ in three tandem repeats from the single copy large invasion plasmid, and the fusion protein is detectable and quantifiable in ELISA-based assays. We could detect antibodies against both LTB and ST after 3 times immunization of mice with ShigETEC. The induced LTB antibodies were functional and inhibited the binding of LT to GM1 ganglioside and LT toxicity to colon cells. Upon intranasal immunization with ShigETEC, we were not able to demonstrate ST neutralization with the serum probably due to the low levels of anti-ST antibodies. However, anti-ST_N12S_ antibodies showed ST-neutralizing capacity after parenteral immunization of mice with the recombinantly expressed LTB-ST_N12S_ fusion protein confirming the potential of the fusion protein to induce toxin-neutralizing antibodies. Importantly, antibodies raised against the mutant ST_N12S_ were capable of neutralizing wild-type ST as efficiently as antibodies raised against wild-type ST.

A long existing challenge in Shigella vaccine development is the balance between reactogenicity and antigenicity of live attenuated oral Shigella vaccines. Insufficient attenuation of other vaccine candidates has limited the vaccine dose that could be administered without inducing reactogenicity, and low doses of vaccine have resulted in low level of immune response insufficient to achieve the desired level of efficacy [7]. 

Because of the non-invasive nature of ShigETEC and the previous experiences with VADIZEN, we expect that high doses of ShigETEC can be used (10^11^ CFU/dose) without induction of reactogenicity. However, it is notable that two enterotoxins of *Shigella flexneri* 2, ShET-1 and 2 are implicated in pre-dysenteric watery diarrhea of shigellosis, and isogenic ShET1/2 defective oral vaccine strains have improved safety in Phase I clinical studies [15,37]. Thus, to further reduce the risk of potential side effects at such high doses of ShigETEC, the *setBA* gene was deleted. This accomplished two things. First, the ShET-1 enterotoxin of *S. flexneri* 2 was deleted. Second, *setBA* deletion also eliminates the virulence gene *pic* from the complimentary DNA strand of the locus, hence further attenuating the vaccine strain [38]. Furthermore, the ShET-2 toxin is non-functional in ShigETEC, since it requires an intact type III secretion system, which has been inactivated in ShigETEC. 

We demonstrated that immunization with ShigETEC induced high level of anti-Shigella antibodies already after one-time vaccination. However, the induction of anti-LTB and anti-ST antibodies required multiple immunizations. Based on these data we expect to use a multiple-dose regiment to provide protection against ETEC. ShigETEC is currently being tested in Phase I clinical trial.

## 5. Conclusions

The novel rough and non-invasive vaccine strain ShigETEC has the potential to be a combination vaccine providing protection against potentially all Shigella species and serotypes as well as against ETEC infection. The nature of the vaccine consisting of only one live, attenuated bacterial strain with oral application poses a cost-effective approach for broad coverage of the most vulnerable populations in low- and middle-income countries.

## 6. Patents

Eveliqure Biotechnologies GmbH holds patents and patent applications related to this project.

## Figures and Tables

**Table 1:** Overview of the genetic manipulations introduced to the *Shigella flexneri* 2a 2547T strain to generate the ShigETEC vaccine.

Genetic Manipulation	Location of wt Gene	Phenotypic Change
Deletions:		
*rfbF*	chromosome	Rough, lacking LPS O-antigen
*ipaBC*	invasion plasmid	Non-invasive
*setBA*	chromosome	ShET-1 and Pic defective
*infA*	chromosome	Trans-positioned to the invasion plasmid for plasmid stabilization
Insertions:		
infA-3xLTB-ST_N12S_	n.a.	Stable invasion plasmid, expression of ETEC toxoid antigens

**Table 2:** Severity scores of eyes infected with either ShigETEC or the parental wild-type *Shigella flexneri* 2a 2457T strain in the Guinea pig keratoconjunctivitis model (Serény test).

	Experiment #1								Experiment #2							
	Wild-Type				ShigETEC				Wild-Type				ShigETEC			
	10^6^	10^7^	10^8^	10^9^	10^6^	10^7^	10^8^	10^9^	10^6^	10^7^	10^8^	10^9^	10^6^	10^7^	10^8^	10^9^
Day 1	0	1	1	1	0	0	0	0	0	0	1	1	0	0	0	0
Day 2	0	1	1	2	0	0	0	0	0	0	1	1	0	0	0	0
Day 3	0	1	2	2	0	0	0	0	0	0	1	2	0	0	0	0
Day 4	0	2	2	3	0	0	0	0	0	0	2	3	0	0	0	0
Day 5	0	3	3	3	0	0	0	0	0	0	2	3	0	0	0	0
Day 6	0	4	4	4	0	0	0	0	0	0	2	4	0	0	0	0
